# Synthesis and crystal structure of *γ*-SrNCN at 38 GPa

**DOI:** 10.1107/S2056989026006080

**Published:** 2026-06-12

**Authors:** Lukas Brüning, Georg Krach, Pascal Lennert Jurzick, Wolfgang Schnick, Maxim Bykov

**Affiliations:** a Institute for Inorganic and Analytical Chemistry, Goethe University Frankfurt, Max-von-Laue-Strasse 7, 60438 Frankfurt am Main, Germany; b Department of Chemistry, University of Munich (LMU), Butenandtstrasse 5-13 (D), 81377 Munich, Germany

**Keywords:** crystal structure, high-pressure high-temperature synthesis, nitrides, polymorphism, carbodi­imide

## Abstract

*γ*-Strontium carbodi­imide, γ-SrNCN, was synthesized from a mixture of strontium subnitride (Sr_2_N) and tetra­cyano­ethyl­ene (C_6_N_4_) at 38 (3) GPa in a laser-heated diamond anvil cell. Its crystal structure was solved and refined using synchrotron single-crystal X-ray diffraction. The new polymorph crystallizes in space group *I*4/*mcm* (No. 140), where the Sr^2+^ and NCN^2−^ packing can be derived from the CsCl (B2) structure type. *γ*-SrNCN (*tI*16-SrNCN) is isostructural to *tI*16-BaNCN and represents the first high-pressure polymorph of SrNCN.

## Abstract 2

*γ*-SrNCN was synthesized from a mixture of strontium subnitride (Sr_2_N) and tetra­cyano­ethyl­ene (C_6_N_4_) at 38 (3) GPa in a laser-heated diamond anvil cell. The new polymorph crystallizes in the space group *I*4/*mcm* (No. 140), where the Sr^2+^ and NCN^2−^ packing can be derived from the CsCl (B2) structure type.

## 1. Chemical context

Inorganic carbodi­imide salts are an inter­esting and well-established class of materials that can exhibit exciting optical, magnetic, and catalytic properties (Corkett *et al.*, 2024 ▸). Two polymorphs of SrNCN have been reported thus far. The first characterized polymorph of strontium carbodi­imide, *α*-SrNCN (*oP*16-SrNCN, NaSCN structure type), was synthesized through a reaction of melamine (C_3_N_6_H_6_) with strontium subnitride (Sr_2_N) at 1123 K (Berger & Schnick, 1994 ▸). Polycrystalline *β*-SrNCN (*hR*12-SrNCN, *β*-NaN_3_ structure type) was synthesized from SrCO_3_ in liquid NH_3_ (Wissmann, 2001 ▸), while crystals suitable for single-crystal X-ray diffraction were obtained by heating reactive fluxes of SrI_2_, NaCN and NaN_3_ (2:1:1) at 1073 K, followed by slow cooling (Liao & Dronskowski, 2004 ▸). Krings *et al.* (2010 ▸) further expanded the range of synthetic routes to both *α*- and *β*-SrNCN and showed that *β*-SrNCN is the ground-state polymorph. *α*-SrNCN is used as a host lattice for Eu^2+^ doping, yielding an efficient orange-emitting phosphor (Krings *et al.*, 2011 ▸). Here, we report the synthesis of a high-pressure polymorph, *γ*-SrNCN (*tI*16-SrNCN), from a mixture of strontium subnitride (Sr_2_N) and tetra­cyano­ethyl­ene (C_6_N_4_) at 38 (3) GPa. *γ*-SrNCN is isostructural to *tI*16-BaNCN, which is produced in a reaction of BaCO_3_ in liquid NH_3_ at ambient pressure and an elevated temperature of 1173 K (Masubuchi *et al.*, 2018 ▸). *tI*16-BaNCN remains stable upon compression up to 23 GPa. At higher pressures, it undergoes a symmetry-lowering phase transition to *mC*16-BaNCN, driven predominantly by tilting of the NCN^2−^ anions (Masubuchi *et al.*, 2022 ▸; Yamamoto *et al.*, 2026 ▸). There are a few other examples of high-pressure studies of carbodi­imides (Solozhenko *et al.*, 2004 ▸; Glaser *et al.*, 2008 ▸; Möller *et al.*, 2018 ▸; Meinerzhagen *et al.*, 2024 ▸; Yang *et al.*, 2024 ▸). Furthermore, high-pressure and high-temperature conditions have proven the feasibility of synthesizing ternary nitridocarbonates with increased coordination numbers of three and four for carbon (Brüning *et al.*, 2023 ▸; Aslandukov *et al.*, 2024 ▸).

## 2. Structural commentary

**Figure 1:**
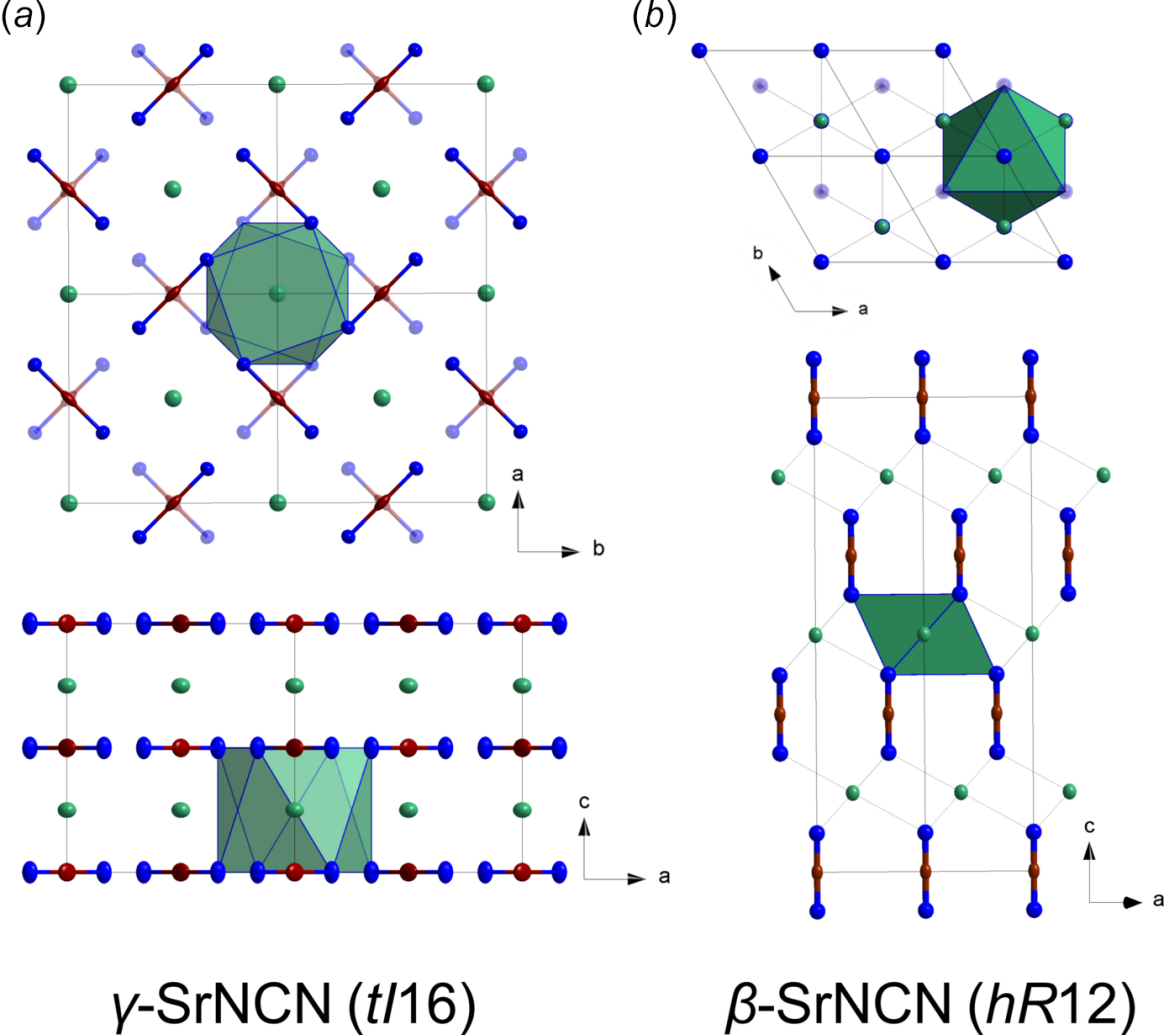
Crystal structure representations of (*a*) *γ*-SrNCN and (*b*) *β*-SrNCN along different crystallographic axes. Displacement ellipsoids are drawn at the 70% probability level. Sr, C and N atoms are colored green, brown and blue, respectively. Semitransparent atoms imply the next layer.

*γ*-SrNCN crystallizes in the space group *I*4/*mcm* (No. 140, KN_3_ structure type), where Sr, C, and N occupy the Wyckoff positions 4*a* (site symmetry 422), *4d* (site symmetry *m.mm*) and 8*h* (site symmetry *m*.2*m*), respectively. The bond lengths and geometry of the NCN^2−^ anion are only weakly affected by pressure, with *d*(C—N) = 1.222 (13) Å in the reported structure compared to *d*(C—N) = 1.232 (5) Å in *β*-SrNCN at ambient pressure. In contrast to *α*-SrNCN and *β*-SrNCN, where Sr is sixfold coordinated in an octa­hedral environment with *d*(Sr—N) = 2.600 (8) − 2.657 (8) Å, *γ*-SrNCN features Sr in an eightfold tetra­gonal anti­prismatic coordination with *d*(Sr—N) = 2.460 (4) Å. Similar to *β*-SrNCN, *γ*-SrNCN is built from stacked layers of Sr^2+^ cations and linear NCN^2−^ anions (Fig. 1 ▸). The main difference between the polymorphs arises from the rearrangement of NCN^2−^ units: in *γ*-SrNCN, they are oriented parallel to the Sr^2+^ layers rather than perpendicular to them like in *β*-SrNCN.

Within the NCN^2−^ layers, each linear unit is rotated by 90° in the *ab* plane with respect to the corresponding unit within the adjacent layer. Treating the NCN^2−^ unit as a single pseudo-atom would result in an octa­hedral coordination for both Sr and NCN^2−^ in *β*-SrNCN, while the coordination would be cubic for *γ*-SrNCN. Therefore, *β*-SrNCN can be derived from the NaCl (B1) structure type and *γ*-SrNCN packing follows the CsCl (B2) structure type, consistent with the pressure-coordination rule. A parallel can be drawn to the B1 to B2 phase transition in NaCl at 30 GPa (Bassett *et al.*, 1968 ▸) and to the polymorphism of NaN_3_, for which the isostructural high-pressure polymorph *tI*16-NaN_3_ was described (Pulham *et al.*, 2014 ▸). The results also align well with the pressure homologue rule, since CsCl (B2) packing can be achieved in *tI*16-BaNCN at ambient pressure (Masubuchi *et al.*, 2018 ▸).

## 3. Synthesis and crystallization

Strontium subnitride (Sr_2_N) was synthesized via direct reaction of strontium metal with N_2_ gas at 1273 K, as described in the literature (Reckeweg & DiSalvo, 2002 ▸). A 20 µm piece of Sr_2_N was embedded in tetra­cyano­ethyl­ene (C_6_N_4_, Thermo Fischer, purity > 98%), compressed to 38 (3) GPa and laser-heated in a diamond anvil cell (BX90 body design; Boehler–Almax type diamonds with a conical aperture of 70° and 200 µm culet size) with a Nd:YAG laser (λ = 1064 nm, T > 1500 K, 4 s heating time). The same synthesis approach and detailed description of data evaluation can be found in previous works on ternary nitridocarbonates (Brüning *et al.*, 2023 ▸, 2025 ▸; Ranieri *et al.*, 2025 ▸; Jurzick *et al.*, 2026 ▸). Pressure was determined using the pressure–frequency relationship of the stressed diamond Raman band (Akahama & Kawamura, 2006 ▸). The reaction product, *γ*-SrNCN, was polycrystalline with submicron grain sizes, as indicated by XRD mapping of the sample chamber.

## 4. Refinement

**Figure 2:**
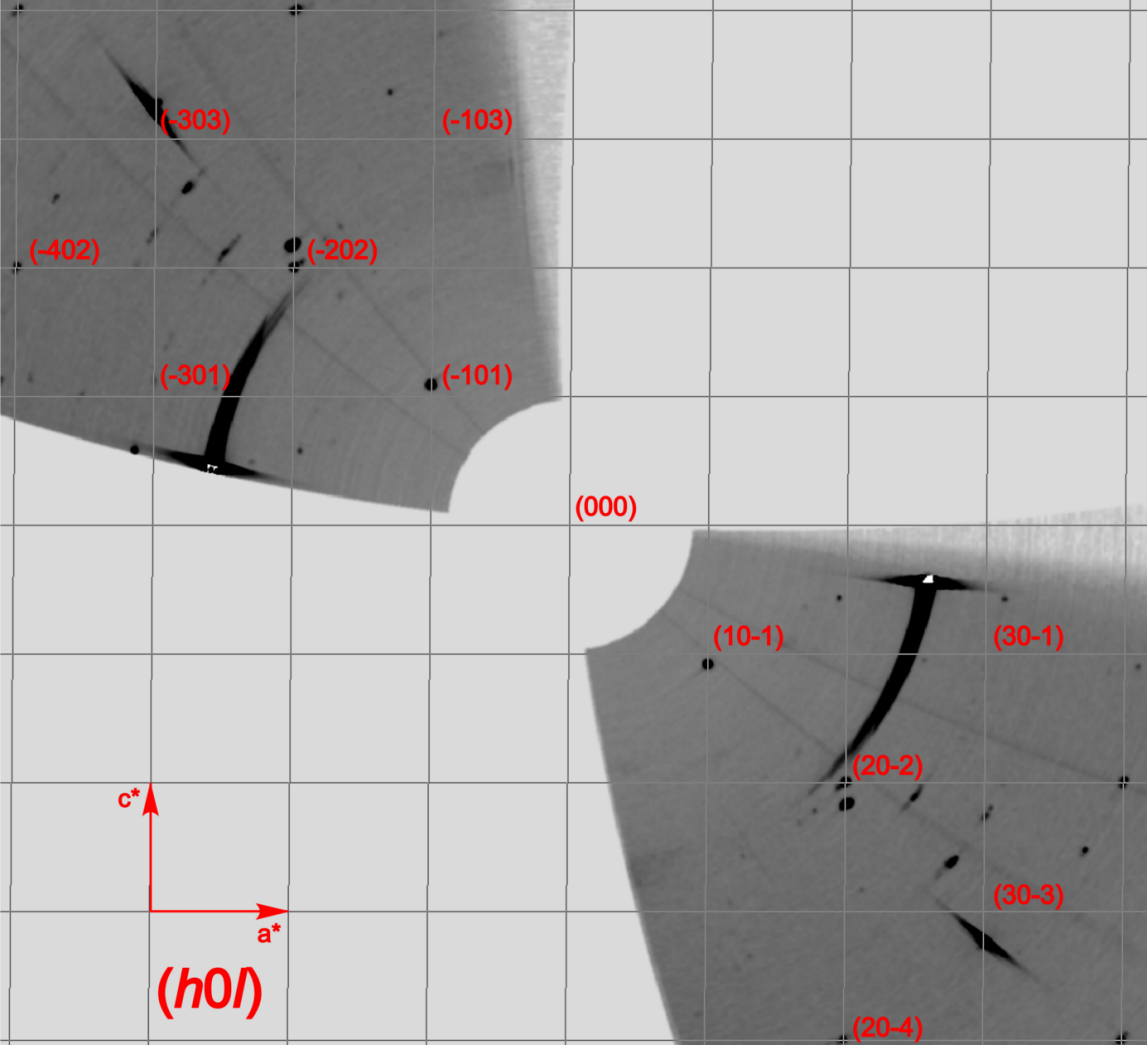
(*h*0*l*)-reciprocal lattice plane of *γ*-SrNCN (No. 140 *I*4/*mcm*) at 38 (3) GPa with indexed reflections fulfilling (*hkl*): *h* + *k* + *l* = 2*n* for *I*-centering and (0*kl*): *k*,*l* = 2*n* [≡ (*h*0*l*): *h*,*l* = 2*n*] for a *c*-glide plane perpendicular to [100] (≡ [010]).

Crystal data, data collection and structure refinement details are summarized in Table 1 ▸. The reaction product was studied by means of synchrotron single-crystal X-ray diffraction at the extreme conditions beamline P02.2 at Deutsches Elektronen Synchrotron (PetraIII, DESY). The X-ray beam had a full width at half maximum of ∼2 µm and a wavelength of 0.2908 Å, and the X-ray diffraction data were measured using a PerkinElmer XRD1621 2D flat panel detector. To obtain single-crystal datasets, the diamond anvil cell was rotated around the vertical ω axis within a range of ±32°. Diffraction data were acquired in 0.5° *ω* steps with an exposure time of 4s per °. Data reduction was performed using *CrysAlis^PRO^* software package. Since the dataset contains diffraction data from multiple crystallites with different orientations, the algorithm DaFi (Aslandukov *et al.*, 2022 ▸) was used to group and extract the orientation matrices from individual crystalline domains. The most prominent domain was used for integration and the resulting *hkl* file was then used for structure solution with *SHELXT* (Sheldrick 2015*a* ▸) and refinement with *SHELXL* (Sheldrick 2015*b* ▸) within the *OLEX2-1.5* inter­face (Dolomanov *et al.*, 2009 ▸). The limited opening of the diamond anvil cell results in reduced completeness of the datasets. As a standard procedure, we carefully examine reconstructed precession images of each dataset to check for missing superlattice reflections and to cross-check the choice of the space-group symmetry. In the case of *γ*-SrNCN, the systematic absences are consistent with the *I*4/*mcm* space group. It can be shown that (0*kl*): *k*,*l* = 2*n* [≡(*h*0*l*): *h,l* = 2*n*] for a *c*-glide plane perpendicular to [100] (≡[010]) in a tetra­gonal body-centered Bravais lattice holds (Fig. 2 ▸).

## Supplementary Material

Crystal structure: contains datablock(s) I. DOI: 10.1107/S2056989026006080/meu2002sup1.cif

Structure factors: contains datablock(s) I. DOI: 10.1107/S2056989026006080/meu2002Isup2.hkl

CCDC reference: 2560951

Additional supporting information:  crystallographic information; 3D view; checkCIF report

## Figures and Tables

**Table 1:** Experimental details

Crystal data	
Chemical formula	SrNCN
*M*_r_	127.65
Crystal system, space group	Tetragonal, *I*4/*mcm*
Temperature (K)	293
*a*, *c* (Å)	5.311 (2), 5.790 (6)
*V* (Å^3^)	163.3 (2)
*Z*	4
Radiation type	Synchrotron, λ = 0.2908 Å
μ (mm^−1^)	3.01
Crystal size (mm)	0.001 × 0.001 × 0.001

Data collection	
Diffractometer	Customized ω-circle diffractometer
Absorption correction	Multi-scan (*CrysAlis PRO*; Rigaku OD, 2025 ▸)
*T*_min_, *T*_max_	0.150, 1.000
No. of measured, independent and observed [*I* > 2σ(*I*)] reflections	366, 109, 70
*R*_int_	0.072
(sin θ/λ)_max_ (Å^−1^)	1.006

Refinement	
*R*[*F*^2^ > 2σ(*F*^2^)], *wR*(*F*^2^), *S*	0.060, 0.156, 1.15
No. of reflections	109
No. of parameters	10
Δρ_max_, Δρ_min_ (e Å^−3^)	1.97, −1.69

Computer programs: *CrysAlis PRO* (Rigaku OD, 2025 ▸), *SHELXT2014/5* (Sheldrick, 2015*a* ▸), *SHELXL2014/7* (Sheldrick, 2015*b* ▸) and *OLEX2* (Dolomanov *et al.*, 2009 ▸).

## supplementary crystallographic information

### γ-Strontium carbodiimide Crystal data

**Table:**

SrNCN	*D*_x_ = 5.191 Mg m^−^^3^
*M**_r_* = 127.65	Synchrotron radiation, λ = 0.2908 Å
Tetragonal, *I*4/*m**c**m*	Cell parameters from 94 reflections
*a* = 5.311 (2) Å	θ = 2.2–14.7°
*c* = 5.790 (6) Å	µ = 3.01 mm^−^^1^
*V* = 163.3 (2) Å^3^	*T* = 293 K
*Z* = 4	Irregular, dull dark gray
*F*(000) = 232	0.001 × 0.001 × 0.001 mm

### γ-Strontium carbodiimide Data collection

**Table:**

Customized ω-circle diffractometer	109 independent reflections
Radiation source: synchrotron, PETRAIII, Beamline P02.2	70 reflections with *I* > 2σ(*I*)
Synchrotron monochromator	*R*_int_ = 0.072
Detector resolution: 5.0 pixels mm^-1^	θ_max_ = 17.0°, θ_min_ = 3.6°
ω scans	*h* = −7→7
Absorption correction: multi-scan (CrysAlisPro; Rigaku OD, 2025)	*k* = −8→7
*T*_min_ = 0.150, *T*_max_ = 1.000	*l* = −7→8
366 measured reflections	

### γ-Strontium carbodiimide Refinement

**Table:**

Refinement on *F*^2^	0 restraints
Least-squares matrix: full	Primary atom site location: dual
*R*[*F*^2^ > 2σ(*F*^2^)] = 0.060	*w* = 1/[σ^2^(*F*_o_^2^) + (0.0651*P*)^2^] where *P* = (*F*_o_^2^ + 2*F*_c_^2^)/3
*wR*(*F*^2^) = 0.156	(Δ/σ)_max_ < 0.001
*S* = 1.15	Δρ_max_ = 1.97 e Å^−^^3^
109 reflections	Δρ_min_ = −1.69 e Å^−^^3^
10 parameters	

### γ-Strontium carbodiimide Special details

**Table:**

Experimental. X-ray diffraction was measured at 38 (3) GPa on synthesis products, produced by laser-heating in a diamond anvil cell with an opening angle of 70°. Tetracyanoethylene was used as the pressure-transmitting medium. Pressure was determined using the pressure-frequency relationship of the stressed diamond Raman band.
Geometry. All esds (except the esd in the dihedral angle between two l.s. planes) are estimated using the full covariance matrix. The cell esds are taken into account individually in the estimation of esds in distances, angles and torsion angles; correlations between esds in cell parameters are only used when they are defined by crystal symmetry. An approximate (isotropic) treatment of cell esds is used for estimating esds involving l.s. planes.

### γ-Strontium carbodiimide Fractional atomic coordinates and isotropic or equivalent isotropic displacement parameters (Å^2^)

**Table:**

	*x*	*y*	*z*	*U*_iso_*/*U*_eq_	
Sr01	0.5000	0.5000	0.2500	0.0118 (5)	
C1	0.5000	0.0000	0.0000	0.012 (3)	
N1	0.3373 (18)	0.1627 (18)	0.0000	0.013 (2)	

### γ-Strontium carbodiimide Atomic displacement parameters (Å^2^)

**Table:**

	*U*^11^	*U*^22^	*U*^33^	*U*^12^	*U*^13^	*U*^23^
Sr01	0.0132 (6)	0.0132 (6)	0.0092 (11)	0.000	0.000	0.000
C1	0.012 (5)	0.012 (5)	0.013 (11)	−0.008 (6)	0.000	0.000
N1	0.009 (3)	0.009 (3)	0.019 (8)	0.001 (4)	0.000	0.000

### γ-Strontium carbodiimide Geometric parameters (Å, º)

**Table:**

Sr01—Sr01^i^	2.895 (3)	C1—Sr01^ix^	3.0245 (11)
Sr01—Sr01^ii^	2.895 (3)	C1—Sr01^x^	3.0245 (11)
Sr01—C1^iii^	3.0245 (11)	C1—Sr01^xi^	3.0245 (11)
Sr01—C1	3.0245 (11)	C1—Sr01^vii^	3.0245 (11)
Sr01—N1^iv^	2.460 (4)	C1—Sr01^xii^	3.0245 (11)
Sr01—N1^v^	2.460 (4)	C1—Sr01^i^	3.0245 (11)
Sr01—N1	2.460 (4)	C1—Sr01^xiii^	3.0245 (11)
Sr01—N1^vi^	2.460 (4)	C1—N1	1.222 (13)
Sr01—N1^i^	2.460 (4)	C1—N1^xii^	1.222 (13)
Sr01—N1^vii^	2.460 (4)	N1—Sr01^xi^	2.460 (4)
Sr01—N1^iii^	2.460 (4)	N1—Sr01^vii^	2.460 (4)
Sr01—N1^viii^	2.460 (4)	N1—Sr01^i^	2.460 (4)
			
Sr01^ii^—Sr01—Sr01^i^	180.0	N1^viii^—Sr01—N1^vi^	86.5 (5)
Sr01^ii^—Sr01—C1	118.59 (3)	N1^iv^—Sr01—N1^v^	149.1 (6)
Sr01^i^—Sr01—C1^iii^	118.59 (3)	N1^iii^—Sr01—N1^vii^	69.74 (7)
Sr01^i^—Sr01—C1	61.41 (3)	N1—Sr01—N1^vi^	69.74 (7)
Sr01^ii^—Sr01—C1^iii^	61.41 (3)	N1^iv^—Sr01—N1^iii^	80.5 (2)
C1—Sr01—C1^iii^	180.0	Sr01—C1—Sr01^xi^	103.24 (2)
N1^viii^—Sr01—Sr01^i^	126.04 (7)	Sr01^xii^—C1—Sr01^vii^	103.24 (2)
N1^iv^—Sr01—Sr01^ii^	126.04 (7)	Sr01^vii^—C1—Sr01^x^	180.0
N1^iii^—Sr01—Sr01^ii^	53.96 (7)	Sr01^xi^—C1—Sr01^vii^	57.19 (5)
N1—Sr01—Sr01^i^	53.96 (7)	Sr01^ix^—C1—Sr01^x^	57.19 (5)
N1—Sr01—Sr01^ii^	126.04 (7)	Sr01^xiii^—C1—Sr01^x^	103.24 (2)
N1^iv^—Sr01—Sr01^i^	53.96 (7)	Sr01^ix^—C1—Sr01^vii^	122.81 (5)
N1^vi^—Sr01—Sr01^i^	53.96 (7)	Sr01^ix^—C1—Sr01^xi^	180.0
N1^i^—Sr01—Sr01^i^	53.96 (7)	Sr01^i^—C1—Sr01^vii^	103.24 (2)
N1^viii^—Sr01—Sr01^ii^	53.96 (7)	Sr01—C1—Sr01^x^	103.24 (2)
N1^i^—Sr01—Sr01^ii^	126.04 (7)	Sr01^xii^—C1—Sr01^x^	76.76 (2)
N1^vii^—Sr01—Sr01^ii^	53.96 (7)	Sr01—C1—Sr01^xii^	180.0
N1^vi^—Sr01—Sr01^ii^	126.04 (7)	Sr01—C1—Sr01^ix^	76.76 (2)
N1^vii^—Sr01—Sr01^i^	126.04 (7)	Sr01—C1—Sr01^vii^	76.76 (2)
N1^v^—Sr01—Sr01^i^	126.04 (7)	Sr01^xi^—C1—Sr01^xiii^	103.24 (2)
N1^v^—Sr01—Sr01^ii^	53.96 (7)	Sr01^i^—C1—Sr01^x^	76.76 (2)
N1^iii^—Sr01—Sr01^i^	126.04 (7)	Sr01^xii^—C1—Sr01^xiii^	57.19 (5)
N1^i^—Sr01—C1	110.97 (18)	Sr01^xiii^—C1—Sr01^vii^	76.76 (2)
N1^vii^—Sr01—C1^iii^	91.5 (2)	Sr01—C1—Sr01^i^	57.19 (5)
N1^iii^—Sr01—C1	157.1 (3)	Sr01^xi^—C1—Sr01^xii^	76.76 (2)
N1^iii^—Sr01—C1^iii^	22.9 (3)	Sr01^ix^—C1—Sr01^i^	103.24 (2)
N1^viii^—Sr01—C1^iii^	53.8 (3)	Sr01^xi^—C1—Sr01^x^	122.81 (5)
N1^vii^—Sr01—C1	88.5 (2)	Sr01^xi^—C1—Sr01^i^	76.76 (2)
N1^i^—Sr01—C1^iii^	69.03 (18)	Sr01—C1—Sr01^xiii^	122.81 (5)
N1^vi^—Sr01—C1	53.8 (3)	Sr01^xii^—C1—Sr01^i^	122.81 (5)
N1^viii^—Sr01—C1	126.2 (3)	Sr01^xiii^—C1—Sr01^i^	180.0
N1^vi^—Sr01—C1^iii^	126.2 (3)	Sr01^ix^—C1—Sr01^xiii^	76.76 (2)
N1—Sr01—C1^iii^	157.1 (3)	Sr01^ix^—C1—Sr01^xii^	103.24 (2)
N1^v^—Sr01—C1	69.03 (18)	N1^xii^—C1—Sr01^vii^	128.380 (11)
N1—Sr01—C1	22.9 (3)	N1^xii^—C1—Sr01^i^	128.380 (11)
N1^iv^—Sr01—C1^iii^	88.5 (2)	N1—C1—Sr01^xii^	128.380 (11)
N1^iv^—Sr01—C1	91.5 (2)	N1—C1—Sr01^x^	128.380 (11)
N1^v^—Sr01—C1^iii^	110.97 (18)	N1^xii^—C1—Sr01^ix^	51.620 (11)
N1^viii^—Sr01—N1^i^	80.5 (2)	N1^xii^—C1—Sr01^xii^	51.620 (11)
N1^vi^—Sr01—N1^v^	80.5 (2)	N1^xii^—C1—Sr01	128.380 (11)
N1—Sr01—N1^v^	86.5 (5)	N1—C1—Sr01^vii^	51.620 (11)
N1^i^—Sr01—N1^v^	138.9 (5)	N1—C1—Sr01^xiii^	128.380 (11)
N1^viii^—Sr01—N1^iii^	69.74 (7)	N1—C1—Sr01^i^	51.620 (11)
N1^viii^—Sr01—N1^vii^	107.91 (13)	N1^xii^—C1—Sr01^xi^	128.380 (11)
N1^vii^—Sr01—N1^v^	69.74 (7)	N1—C1—Sr01^ix^	128.380 (11)
N1—Sr01—N1^i^	107.91 (13)	N1^xii^—C1—Sr01^x^	51.620 (11)
N1^viii^—Sr01—N1^iv^	138.9 (5)	N1—C1—Sr01	51.620 (11)
N1—Sr01—N1^iv^	69.74 (7)	N1^xii^—C1—Sr01^xiii^	51.620 (11)
N1^viii^—Sr01—N1^v^	69.74 (7)	N1—C1—Sr01^xi^	51.620 (11)
N1^iv^—Sr01—N1^i^	69.74 (7)	N1^xii^—C1—N1	180.0 (13)
N1^iv^—Sr01—N1^vii^	86.5 (5)	Sr01—N1—Sr01^i^	72.09 (13)
N1^iii^—Sr01—N1^v^	107.91 (13)	Sr01^xi^—N1—Sr01	149.1 (6)
N1^iv^—Sr01—N1^vi^	107.91 (13)	Sr01^xi^—N1—Sr01^i^	99.5 (2)
N1^iii^—Sr01—N1^i^	86.5 (5)	Sr01^xi^—N1—Sr01^vii^	72.09 (13)
N1^iii^—Sr01—N1^vi^	149.1 (6)	Sr01—N1—Sr01^vii^	99.5 (2)
N1^viii^—Sr01—N1	149.1 (6)	Sr01^vii^—N1—Sr01^i^	149.1 (6)
N1^vii^—Sr01—N1^vi^	138.9 (5)	C1—N1—Sr01^i^	105.5 (3)
N1^vii^—Sr01—N1^i^	149.1 (6)	C1—N1—Sr01^xi^	105.5 (3)
N1^i^—Sr01—N1^vi^	69.74 (7)	C1—N1—Sr01^vii^	105.5 (3)
N1—Sr01—N1^iii^	138.9 (5)	C1—N1—Sr01	105.5 (3)
N1—Sr01—N1^vii^	80.5 (2)		
			
Sr01—C1—N1—Sr01^xi^	180.000 (1)	Sr01^ix^—C1—N1—Sr01	0.000 (1)
Sr01^xii^—C1—N1—Sr01^vii^	75.25 (6)	Sr01^xiii^—C1—N1—Sr01^xi^	−75.25 (6)
Sr01^xiii^—C1—N1—Sr01^i^	180.0	Sr01^xi^—C1—N1—Sr01	180.0
Sr01^xi^—C1—N1—Sr01^vii^	75.25 (6)	Sr01^xiii^—C1—N1—Sr01^vii^	0.0
Sr01—C1—N1—Sr01^i^	75.25 (6)	Sr01^xii^—C1—N1—Sr01	180.0
Sr01^xii^—C1—N1—Sr01^i^	−104.75 (6)	Sr01^i^—C1—N1—Sr01^xi^	104.75 (6)
Sr01^ix^—C1—N1—Sr01^vii^	−104.75 (6)	Sr01^xiii^—C1—N1—Sr01	104.75 (6)
Sr01^ix^—C1—N1—Sr01^xi^	180.0	Sr01^ix^—C1—N1—Sr01^i^	75.25 (6)
Sr01^i^—C1—N1—Sr01^vii^	180.0	Sr01^i^—C1—N1—Sr01	−75.25 (6)
Sr01^x^—C1—N1—Sr01^vii^	180.0	Sr01^vii^—C1—N1—Sr01^xi^	−75.25 (6)
Sr01^xi^—C1—N1—Sr01^i^	−104.75 (6)	Sr01^vii^—C1—N1—Sr01	104.75 (6)
Sr01^xii^—C1—N1—Sr01^xi^	0.000 (1)	Sr01^vii^—C1—N1—Sr01^i^	180.0
Sr01^x^—C1—N1—Sr01^i^	0.000 (1)	Sr01^x^—C1—N1—Sr01	−75.25 (6)
Sr01—C1—N1—Sr01^vii^	−104.75 (6)	Sr01^x^—C1—N1—Sr01^xi^	104.75 (6)

Symmetry codes: (i) −*x*+1, −*y*+1, −*z*; (ii) −*x*+1, −*y*+1, −*z*+1; (iii) −*y*+1/2, *x*+1/2, *z*+1/2; (iv) *y*, −*x*+1, −*z*; (v) *y*+1/2, −*x*+1/2, −*z*+1/2; (vi) −*y*+1, *x*, *z*; (vii) −*x*+1/2, −*y*+1/2, −*z*+1/2; (viii) *x*+1/2, *y*+1/2, *z*+1/2; (ix) −*x*+3/2, −*y*+1/2, −*z*+1/2; (x) *x*+1/2, *y*−1/2, *z*−1/2; (xi) *x*−1/2, *y*−1/2, *z*−1/2; (xii) −*x*+1, −*y*, −*z*; (xiii) *x*, *y*−1, *z*.
